# Using Co-Production to Optimise Support to Address Behaviours of Concern in the STOMP Program

**DOI:** 10.1192/bjo.2026.11420

**Published:** 2026-06-30

**Authors:** Shaan Kotecha, Ivy Lim, Helena Keep, Laura Checkley, Ian Hall

**Affiliations:** East London NHS Foundation Trust, London, United Kingdom

## Abstract

**Aims:**

People with intellectual disability are frequently prescribed psychotropic medications for challenging behaviour, despite guidance advocating for holistic and non-pharmacological Multidisciplinary Team (MDT) support to understand and address the underlying causes of challenging behaviours. Approximately 17% of adults with intellectual disabilities receive antipsychotics for symptomatic management in the absence of mental illness, a rate 16 times higher than the general population. STOMP (Stopping The Over Medication of People with Intellectual Disabilities and/or Autism) is a national project to reduce inappropriate prescribing. Decreasing psychotropic medications without appropriate alternatives risks destabilisation, and so we wanted to ensure our STOMP pathway provided holistic support tomeet the needs and wishes of service users, their carers and care providers. We used a co-production methodology to achieve this.

**Methods:**

Service users eligible for STOMP review were identified. Semi-structured interviews were conducted with family carers (n=8) and care-provider representatives (n=4). Interview guides were based on STOMP principles and refined by the MDT. Interviews were analysed using content and thematic coding. Findings were reported back to participants and cross-verified using a large language model for research triangulation, and then translated into pathway components during MDT design meetings.

**Results:**

Interviews highlighted shared priorities for reducing challenging behaviour and improving quality of life. Both groups stressed the need for coordinated input from the Integrated Learning Disability Service and primary care, predictable routines, meaningful tasks with supported independence, clear plans for care changes, an allocated worker, and communication support. Participants felt medication provided stability but did not address underlying triggers. They requested more thorough medication reviews with clearer explanations of rationale and side effects. Significant concern was expressed about destabilisation if medication was reduced without reliable alternatives and clear crisis plans. Service improvement priorities included respite provision, timely social care responses, better access to physical healthcare, and tailored staff training.

**Conclusion:**

Co-production identified significant anxieties about medication reduction, but emphasised that it is feasible when reliable alternatives are in place, such as coordinated MDT input, communication support, and clear crisis plans. The analysis showed that good social care support forms a large and essential element of this, including community access,day opportunities and respite provision. Integrated Learning Disability Services (with health and social care working together in the same organisation) can make such a service user focused approach much easier to deliver, which should result in significant improvements in quality of life, and reduced side-effect burden.

